# Design of Interoperable Electronic Health Record (EHR) Application for Early Detection of Lung Diseases Using a Decision Support System by Expanding Deep Learning Techniques

**DOI:** 10.2174/0118743064296470240520075316

**Published:** 2024-06-06

**Authors:** Jagadamba G, Shashidhar R, Vinayakumar Ravi, Sahana Mallu, Tahani Jaser Alahmadi

**Affiliations:** 1 Department of Information Science and Engineering, Siddaganaga Institute of Technology, Tumakuru, Karnataka- 57210, India; 2 Department of Electronics and Communication Engineering, JSS Science and Technology University, Mysuru, Karnataka 570006, India; 3 Center for Artificial Intelligence, Prince Mohammad Bin Fahd University, Khobar, Saudi Arabia; 4 Department of Electronics and Communication Engineering, ATME College of Engineering, Mysore, Karnataka, India; 5 Department of Information Systems, College of Computer and Information Sciences, Princess Nourah Bint Abdulrahman University, P.O. Box 84428, Riyadh 11671, Saudi Arabia

**Keywords:** Lung disease, Electronic medical records (EMR), Electronic health records (EHR), Carcinoma, Interoperability, Decision support system, Deep Learning, Healthcare, Disease prediction, Convolution Neural Network (CNN), Artificial Neural Network (ANN), Cell carcinoma, Adenocarcinoma carcinoma, Squamous cell carcinoma

## Abstract

**Background:**

Electronic health records (EHRs) are live, digital patient records that provide a thorough overview of a person's complete health data. Electronic health records (EHRs) provide better healthcare decisions and evidence-based patient treatment and track patients' clinical development. The EHR offers a new range of opportunities for analyzing and contrasting exam findings and other data, creating a proper information management mechanism to boost effectiveness, quick resolutions, and identifications.

**Aim:**

The aim of this studywas to implement an interoperable EHR system to improve the quality of care through the decision support system for the identification of lung cancer in its early stages.

**Objective:**

The main objective of the proposed system was to develop an Android application for maintaining an EHR system and decision support system using deep learning for the early detection of diseases. The second objective was to study the early stages of lung disease to predict/detect it using a decision support system.

**Methods:**

To extract the EHR data of patients, an android application was developed. The android application helped in accumulating the data of each patient. The accumulated data were used to create a decision support system for the early prediction of lung cancer. To train, test, and validate the prediction of lung cancer, a few samples from the ready dataset and a few data from patients were collected. The valid data collection from patients included an age range of 40 to 70, and both male and female patients. In the process of experimentation, a total of 316 images were considered. The testing was done by considering the data set into 80:20 partitions. For the evaluation purpose, a manual classification was done for 3 different diseases, such as large cell carcinoma, adenocarcinoma, and squamous cell carcinoma diseases in lung cancer detection.

**Results:**

The first model was tested for interoperability constraints of EHR with data collection and updations. When it comes to the disease detection system, lung cancer was predicted for large cell carcinoma, adenocarcinoma, and squamous cell carcinoma type by considering 80:20 training and testing ratios. Among the considered 336 images, the prediction of large cell carcinoma was less compared to adenocarcinoma and squamous cell carcinoma. The analysis also showed that large cell carcinoma occurred majorly in males due to smoking and was found as breast cancer in females.

**Conclusion:**

As the challenges are increasing daily in healthcare industries, a secure, interoperable EHR could help patients and doctors access patient data efficiently and effectively using an Android application. Therefore, a decision support system using a deep learning model was attempted and successfully used for disease detection. Early disease detection for lung cancer was evaluated, and the model achieved an accuracy of 93%. In future work, the integration of EHR data can be performed to detect various diseases early.

## 1 INTRODUCTION

Data and communications technology (DCT) has changed how clinical patient data are collected, stored, used, and disseminated. The transition from paper-based to electronic record-keeping has been ongoing in all fields, and it has been described using a variety of names in the health sector. Automated health records (AHR), computerised patient records (CPR), electronic medical records (EMR), and electronic health records (EHR) are a few examples [1]. According to the World Health Organization and the regional office for the Western Pacific in 2006, “AHR” stands for “Automated health records” and refers to a collection of medical records stored as digital images in a computer. In the middle of the 1990s, when information was stored as pictures on optical plates, these types of health records were used. This solution helped with the space and accessibility problems associated with traditional paper-based records. In addition, patient identification connected the various health data that were described in the computer-based patient record (CPR) for a single patient, either for a single episode or for a more extended period of care (World Health Organization and Regional Office for the Western Pacific 2006). The computer-based patient record (CPR) focuses on features including warnings and prescription orders, and it provides coordinated patient data from various departments like the pharmacy, research facility, radiology, and so on.

A few clinicians utilize the terms EHR and EMR; conversely, the advantages they offer fluctuate incredibly. An EMR (electronic clinical record) is a centralized and non-sharable electronic health record of the individual, and an EHR (electronic health record) is a centralized and shareable record with all other organizations [2, 3]. Electronic medical records (EMRs) are digital versions of all the information typically found in a provider's paper graph, including clinical history, analysis, medicines, immunization dates, sensitivities, lab results, and remarks of the specialists. Online clinical records, or EMRs, are often used by suppliers for diagnosis and treatment. They contain standard clinical data from one supplier's office, patient's clinical history, tests, results, and treatment documented to ensure proper consideration across the supplier's facility. In addition, they make it possible for members of a medical services group to communicate and coordinate for the best patient care [4]. Their normal development started during the 1960s, and they had many advantages over paper documents [5]. However, many pros and cons are always associated with new technology. The advantages of having EMR are presented in Table **1** before proceeding towards EHR.

Table **2** describes a few challenges identified for the implementation and utilization of EMR systems in the healthcare industry.

To overcome the challenges discussed in Table **2**, many solutions were advanced; however, they could not overcome most of the disadvantages of having EMR. Hence, an EHR system evolved in the healthcare industry for continuous care and monitoring of an individual's health status.

### 1.1 Electronic Health Record (EHR)

Electronic health records, or EHRs, are a handy and safe way to save a patient's medical history. All authorized companies/organizations and patients can access and share the electronic health record (EHR), including past clinical history, significant physiological functions, progress notes, analysis of medications, immunization dates, sensitivities, test results, and imaging reports in the form of images. The power of an EHR is in the data it holds. It helps with effective care coordination when its common health data are immediately made available to certified suppliers across practices and health organizations. Clinicians and organisations involved in a patient's care, such as labs, trained professionals, imaging centres, drug stores, crisis centres, and health care school and work environment centres, can receive an EHR [2, 3].

As sharing EMR outside the specific clinical practice is not intended [6, 11-13], electronic health record (EHR) is comprehensive sharable data. The benefits of EHR are growing day by day beyond the limitations. The consolidated discussions on the benefits of EHR can be found in Table **3**.

The turn of events and execution of EHRs include heaps of difficulties. It requires adequate financing and a thoroughly prepared labour supply, including specialists from various regions like IT specialists, well-being advisors, instructors, *etc*. Keeping patient records secure is one of the critical difficulties in the execution of EHRs. There are concerns connected with the abuse of the information base and the danger to network safety. The guaranteed protection and secrecy of the patient's record and admittance to information should be given exclusively to the approved clients [11]. Some actions like secret word-safeguarded information, distributed storage, and encryption can resolve issues connected with the security of the EHRs. Safety efforts like antivirus programming, firewalls, and so on ought to be consolidated and used for information uprightness. Planning an easy-to-use point of interaction is another problematic task. Inadequately planned interaction might prompt diminished time proficiency and low quality of medical care conveyance and can also threaten the patient's security [11, 12].

Some of the challenges are listed in Table **4** and are discussed based on the usability and design concerns of the EHR system.

Among the challenges listed in Table **4**, interoperability and data privacy were considered in the research problem while designing and implementing an EHR system. However, the other challenges, such as cost and usability, were kept out of context for the present discussion.

### 1.2 Why Interoperability in EHR?

As per the Office of the National Coordinator for Health Information Technology (ONC) [13], EHR should have four critical areas of innovation effectively incorporated to be viewed as totally interoperable. These include:

1) Application connection with clients.

2) System correspondence.

3) Information handling and the board.

4) Consumer gadget incorporation.

Nationwide, a large number of government-approved EHR products are in use, each having a different set of clinical terminologies, specific conclusions, and practical capabilities. Due to these differences, it is difficult to create a single, universally interoperable design for information sharing. Since they are frequently meticulously tailored to an organization's particular work process and preferences, not even those EHR frameworks built on the same platform are truly interoperable. The process of interoperability is complex. The phrase relates to more than just the ability to exchange data. The ability to trade and then use the data is a requirement for two EHR systems to be truly interoperable. For this to occur, the message delivered must include information that has been normalised and encoded so that the receiving framework can understand it. However, the lack of standardised data is a problem that has plagued the American healthcare system for a very long time and significantly limits the ability to transmit information electronically for patient consideration in the current situation [4].

### 1.3 Why Data Privacy in EHR?

Customers who provide businesses with their personal information offer to access sensitive data. In the process of sharing, the data may fall into the wrong hands and be used against them. Information protection was created to protect businesses and their employees from [13] security breaches. While consenting to information protection guidelines, the significant data cannot be abused. In health care, privacy laws and a fragmented market have kept the industry from reaping the full potential of AI. Federated learning could allow companies to train a decentralized model collaboratively without sharing confidential medical records. From lung scans to brain MRIs, aggregating medical data and analysing them at scale could lead to privacy breaches, especially when new ways of detecting and treating cancer, among other diseases, are executed [14, 15].

### 1.4 Introduction to Clinical Decision Support System (CDSS) Using Deep Learning

Computer-based CDSS was defined nearly around the 1970s as a decision support system (DSS), and it is intended to improve clinical decisions with designated clinical information, patient data, and other wellness data, advancing the delivery of medical care [4]. In a typical CDSS/DSS, patient-explicit evaluations or descriptions are presented to the physician for consideration when the characteristics of a specific patient are matched to an updated clinical information base. However, the programming is designed to provide immediate guidance for clinical direction. CDSSs are primarily used today at the point of care to allow the physician to combine their knowledge with any data or concepts provided by the CDSS. Progressively, CDSSs are being developed with the capacity to exploit data and perspectives, regardless of how absurd or imperturbable they may seem to individuals.

During the 1970s, CDSS usage was restricted to scholarly pursuits [5]. They stressed the concern of raising lawful issues around the utilization of PCs in medication and doctor independence, and this might be due to the suggestion of a framework with inadequate knowledge of practitioners [5]. Currently, DSS uses web applications or combines them with EHR systems for the automation of supplier requests. They can be controlled using a workstation, tablet, or smartphone, as well as other devices like biometric scanning and wearable health technology. These devices may be able to generate results directly from the device or may connect to EHR databases.

The interoperable EHR system provides reminders, alerts, and assistance for diagnosis and treatment. Hence, EHR systems can be used for the clinical decision support system to identify diseases early. Moreover, they support young doctors also. As there are several techniques for early disease prediction, deep learning is chosen as the essential technique that can be adopted in the CDSS/DSS.

The deep learning (DL) calculations advance straightforwardly from crude information without human direction, giving the advantage of finding inactive connections. The DL handles detailed, crude information using fake brains [16, 17]. The organizations (Artificial Neural Network -ANN; PC programs that look like how a human cerebrum imagines) cycle the information through different “stowed away” layers for perfect learning. Considering its similarity to human reasoning, DL has been depicted as less mechanical than conventional ML. The “deep” in an ANN should have more than one secret layer. These layers are comprised of hubs that join information input with a bunch of coefficients (loads) that enhance or contribute towards the expected result. DL is great for finding complicated structures in high-layered information, such as clinician notes generated by EHRs, clinical and non-clinical information given by patients scanning reports, *etc*. A significant watchfulness in DL is that the secret layers inside ANNs can deliver the results, which are hard to decipher (black box peculiarity where it is hazy how a calculation showed up at a result) [18].

## 2 METHODOLOGY

A cutting-edge improvement in the conventional brain network method is adopted in deep learning, where one can see tremendous advancement as a multi-layered brain network is formed. Faster development of current processing enables a profound understanding of building massively layered brain networks, which is impossible for conventional brain organisations. As a result, deep learning may examine more complicated non-linear examples in the EHR data. The growth in the volume and complexity of information provides yet another explanation for the recent surge in the popularity of deep learning [19,20] and the fact that it can be adopted early for EHR data. Many features of DL have been identified and listed in Table **5**.

From the discussions so far, we can conclude that the following objectives are identified for the proposed system.

1) To achieve an interoperability system with the EHR data.

2) Clinical Decision Support system for prediction of disease through EHR data.

3) To provide quality health care through early prediction.

4) To provide quality decision support for medical practitioners in an efficient manner.

### 2.1 Literature Survey

Many papers were reviewed from 2010 to 2020 in the health care field. The primary focus was on EHR design and implementation issues and deep learning techniques adopted for the prediction of disease using EHR data. The study concentrated on (1) characteristics of DL approaches and organization types, (2) CNN models, (3) difficulties of DL and proposal of other arrangements, (4) utilization of DL, and (5) evaluation of computational methodologies. A detailed study has been presented in 2 sections, namely on 1) EHR and 2) DSS.

#### 2.1.1 Electronic Health Record (EHR)

The Health Information Technology for Economic and Clinical Health Act (HITECH Act), which took effect in 2009 and is known as the key transition from EMR to EHR, is presided over by President Barack Obama [6]. Part of the American Recovery and Reinvestment Act of 2009 concentrated on improving patient care. The main goal of the HITECH Act is to encourage the use of Electronic Health Records and other supporting health IT in the United States [9]. It also analyzes how using various specialized and semantic rules has caused conflicts in conventional EHR, leading to information reconciliation and interoperability issues. The problem of information sharing between two medical care frameworks is also a result of different medical care rules, technology, and dialects. To combat this, the act has proposed two cloud-based models, Open Vista and Cloud Health Information Systems Technology Architecture, for the US clinic to incorporate various health administrations (CHISTAR). The Indian Healthcare System has also attested to the advancement of ICT applications; however, these are primarily limited to a small number of private clinics, such as Max Health [19], Sankara Nethralaya [20], Fortis, Apollo [21], and so on. Significant public emergency clinics, including All India Institute of Medical Sciences (AIIMS) and Postgraduate Institute of Medical Education and Research (PGIMER), have Electronic Medical Records (EMR) set up to transmit patient information to various branches of a comparable group of emergency clinics [22]. The execution covers a wide range of topics, including recruiting and billing, research facilities, and clinical data. The developed EMRs are rarely swapped across similar groups of clinics. In most health care, the patient receives a printout of the records for further consultation during an emergency. It occurs at clinics where the patient's information remains in the same clinic and causes delays in treatments. In these situations, executing the EHR and coordinating all levels of the general healthcare framework becomes crucial so that the generation of patient information is easier and more secure both during and after the point of care [23].

### 2.2 Deep Learning Model-Based Prediction System

In one of the previous studies [14], a methodology to identify the early location of infection called a cellular breakdown in the lungs by handling lungs Computed Tomography (CT) pictures utilizing Image Processing strategies, was proposed. However, in a previous study [24], the creators utilized digit plane cutting, disintegration, and Weiner channel picture handling procedures. These methods were utilized to separate the lung districts from the Computed Tomography (CT) picture. Later, they removed lung areas, which were sectioned utilizing region-developing segmentation calculation methodology. When the division was done, a rule-based e-model was utilized to recognize the dangerous knobs, and its efficiency was found to be 80%. A procedure for the cellular breakdown in the lungs recognition on a CT picture was utilized, and image handling was used. The pre-handling stage in this included picture upgradation, where Gabor channel and Fast Fourier change methods were utilized. Further, picture division watershed calculation was applied, and element extraction of the portioned picture in the later stage was used to determine the region, border, and capriciousness to identify and arrange the lung knobs [25, 26]. A similar procedure was proposed in another study [27] to distinguish the lung knobs and give the idea of utilizing Image Processing and Decision-Making strategies. Most importantly, picture pre-handling was done on the CT images, and the pre-handling was done using contrast upgrade and direct separating methods. Further, the sifted picture was sectioned using a region-developing segmentation process. Further elements like size, region, and variety were fed to the fuzzy framework to utilize the capability of fluffy participation to recognize and arrange lung cancer [16]. Another study [5] indicated the way to deal with the recognition of the cellular breakdown in the lungs utilizing Image handling and Neural Network Techniques. The CT image of the lung was sifted to eliminate Gaussian repetitive sound, and Otsu's limit method was used to divide the picture. The primary elements were extricated and utilized for AI classification. The Support Vector Machine and Artificial Neural Network techniques were utilized to order the information in the CT-filtered image. The SVM technique gave a higher exactness of 95.12%. In a previous study [24], a system was proposed to recognize the cellular breakdown in the lungs from Lung CT pictures utilizing brain organizations and Genetic Algorithms. At first, the pre-handling of the picture was upgraded, and the feature extraction and choice stage were performed on the upgraded picture utilizing the genetic algorithm. Soon after, the Back Propagation Neural Network method was used to make the text picture dangerous or non-carcinogenic. In another study [28], a technique for the division of MRI, CT, and Ultrasound pictures was proposed. Right recognizable proof of neoplastic cell was removed by concentrating on the expected elements in the picture. Ultrasound pictures were utilized to distinguish the legitimacy of the framework. For the component determination, Particle Swarm Optimization (PSO), Genetic Optimization, and SVN were performed, which achieved a precision of around 89.5% with a decrease in bogus positives [28]. In another study [21], a planned framework was proposed for the cellular breakdown in the lungs characterization of CT filters with plain knobs. Thresholding was utilized as an introductory section method that delivered the best lung segmentation. A U-Net prepared on LUNA16 information was utilized to recognize knob delegates. The U-Net results were taken care of in 3D convolutional neural networks, and at last, the CT picture was tainted or not so much for the cellular breakdown in the lungs. The three-dimensional Convolution Neural Network gave a test precision of 86.6%. In their study [25], S Senthil and his co-researchers proposed a way to deal with recognizing the cellular breakdown in the lungs by utilizing brain networks with ideal elements. At first, the data pre-handling was applied to the pictures for the picture upgrade. Then, the improved pictures were prepared and tried using brain organization. At first, Particle Swarm Optimization was applied to separate the highlights of the info pictures. The info test was named carcinogenic or non-dangerous, relying upon the Artificial Neural Network strategy. It was seen that the method gave a precision of 97.8% [18]. S Shashikala introduced a procedure to characterize the growths in the lung as harmless or threatening [29]. The CT checked the picture, which was taken and pre-handled, utilizing the middle channel method. Then, the back-engendering calculation was utilized to prepare the CNN to identify the lung cancers in the CT picture. To prepare the model, lung pictures with various shapes and sizes of malignant tissues were taken, and the CNN-based technique had the option to recognize the presence or nonattendance of dangerous cells with an exactness of 96%. Rohit Y. Bhalerao introduced a way to characterize the info CT filter lung pictures as carcinogenic or noncancerous using the CNN technique [30]. Before preparing the pictures utilizing CNN, the info pictures were switched over completely to Grayscale and were changed into a double arrangement for ease in the processing stages. Later, the pictures were prepared using the CNN model to achieve a general exactness of 94% [20].

In summary, the works of many researchers showed that adopting the CNN methodology will be the better option for recognizing the CT image as carcinogenic or non-cancerous. The literature survey also provided sufficient knowledge for pre-processing the image before applying deep learning techniques.

## 3 PROPOSED SYSTEM

### 3.1 System Design

Theoverviewof theproposed systemis shown in Fig. (**1**) and includes two subsystems: 1) EHR system in an Android application and 2) Prediction system.

EHR systems consist of the health data of the patients, which will be managed in the cloud. The data are extracted from:

1) Various sources, such as doctors, laboratories, and patients. The data are updated only through authorized entries. The authorized entries include patients, doctors, and the laboratory in charge.

2) The updated data are stored in the cloud and retrieved securely from the cloud. The data are collected and stored in the cloud, then transferred to the prediction system.

3) The data received will be in a different format, and the disease prediction system will include a lot of pre-processing. In the prediction system, the raw data are pre-processed and sent to feature selection and classification for prediction.

### 3.2 The Proposed EHR System

The proposed system includes admin, patient, doctor, and hospital modules.

#### 3.2.1 Admin

In the admin module, the admin will have the login credentials, such as email ID and password. The admin will have the privilege of controlling the entire system, including the patient, doctor, and hospital modules. The admin can add/remove doctors, patients, and hospitals as per the health organization's instructions.

#### 3.2.2 Patient

In this module, the patient must register for the EHR application and have login credentials for further processing. After registration, various operations, such as looking for the appropriate specialist and appointment booking and prescriptions, can be updated by the doctors.

#### 3.2.3 Doctor

In this module, the doctor will register for the EHR application and have login credentials. In this, they can view the appointments of the patients and add the prescriptions and tests to be done.

#### 3.2.4 Hospital

In this module, the admin can register the hospital and health care organization to the system. After the registration process, the individual hospital can be registered.

#### 3.2.5 Justification

Regarding the ethical approval and consent to participation there, no such rules were followed:

1. A total of 322 images were considered from Kaggle, so no ethical approval was required, according to our knowledge.

2. The other 14 images were collected by volunteers who did not want to disclose their identity anywhere.

### 3.3 Disease Prediction System

The disease prediction system was developed using 3 modules: a Pre-processing module, a feature selection module, and a classification module.

We selected lung cancer detection using CT scan images in this system. The proposed system can be adapted to learn and predict many diseases at the early stages using the EHR data extracted for the patients. The system proposed was verified by extracting the image data rather than textual data.

The deep learning method was applied to identify cellular breakdown in the lungs, which is examined more meticulously in the underneath area. It was observed that the majority of the work was aimed at identifying these particular lung-related illnesses. At the same time, it was learned that one vital trait of cellular breakdown in the lungs is the presence of pneumonic knobs, strong clusters of tissue that show up in and around the lungs. These knobs should be visible in CT examination pictures and can be harmful or harmless. If they are harmful, they could be malignant. Hence, the prediction of these using CT scans and deep learning is the major concern.

#### 3.3.1 Basic Steps for Lung Disease Detection Using Deep Learning

This section describes the process of using deep learning to separate lung conditions from clinical images [31]. The standard three main processes are image pre-processing, preparation for feature selection, and classification. Lung disease classification often involves classifying a picture into healthy lungs or lungs that have been tainted by illness. The lung infection classifier is often referred to as a model. The cycle of feature extraction is where a brain network learns how to perceive a class of images. It is possible to create a model that can divide images into their individual class names using deep learning [24]. In this way, gathering images of lungs with the ailment to be ordered is the first stage in using deep learning for lung disease location. The next step is to train the neural network to detect illnesses. The final improvement is to create fresh group images. Here, the model shows brand-new images that it had previously hidden, and it predicts the category of those images. A high-level view of the procedure is depicted in Fig. (**2**).

Many image-processing techniques can be used in the medical field to diagnose lung diseases. There are four basic strategies for improving lung cancer diagnosis in CT imaging. Each stage includes different procedures, leading to varying degrees of accuracy in lung cancer detection. Any noise in the lung CT scan image is removed during pre-processing. Second, the image is divided into segments to obtain the Region of Interest (ROI). Thirdly, features, including energy, entropy, and variance, are extracted *via* feature extraction. Finally, a separate classification technique is used on the retrieved characteristics of the lung CT image [32].

##### 3.3.1.1 Image Acquisition

In the first action, the system acquires the CT scan images of the user and uses them as the input. These images are applied to obtain better precision and reduced distortion.

##### 3.3.1.2 Grayscale Conversion

An RGB image is transformed into a grayscale image in this step. Taking the average of the red, green, and blue pixel values for each pixel [28] to obtain the grayscale value is a straightforward technique to convert a colour image 3D array to a grayscale 2D array. This creates an approximate grey colour by combining the lightness or brightness contributions from each colour band.

##### 3.3.1.3 Binarization

By applying a threshold value, grayscale images (whose pixel ranges vary from 0 to 255) are transformed into binary images (whose pixel ranges are (0, 1)). Segmentation in the field of medical imaging, serves as a component of screening. In a previous study [29], it was suggested that copy is divided into a number of useful segments by the segmentation method. The digital image is then broken into numerous portions for use in computerized vision and recognition systems. The fundamental goal of segmentation is to make the delegation of a CT scan simple, change it into something more informative, and make it easier to analyse in detail. Image segmentation is used to detect objects and image boundaries like lines, curves, and other features while removing extraneous information from the image. The segmentation method in the suggested system consists of a few steps [24, 25]. The real image is first converted into an edge-only image and then converted into a dilated and filled image before being finally divided into the left and right lungs.

##### 3.3.1.4 Extracting Features

An important level or phase is feature extraction, which employs tools and algorithms to spot patterns [12]. The segmented output is used as the input of the feature extraction. Area, perimeter, and eccentricity are three scalar quality features that are included in feature extraction.

#### 3.3.2 Implementation of Deep Learning Model in the Proposed System

The wireframes created for the application's perspective are described in this section. Before development, these wireframes are used to describe the user's benefit and acquire a broad notion of how the programmer should seem and behave. The users in the wireframe can upload a CT scan from the homepage. The user will be prompted to upload a raw file and a file with information. The system will obtain the prediction and collect this data *via* a POST request to the backend server.

##### 3.3.2.1 Prediction Page

After the CT scan is uploaded, the system unpacks the raw file the user has uploaded. The key feature of the wireframe is that a user can choose a scan to forecast, and the system will utilize the photos to contribute data to a deep learning model.

Anoverview of thedetectionsystemisgiven inFig. (**3**). The system implementation includes the following steps:

##### 3.3.2.2 Upload CT Scan

The user uses a POST request to upload a metadata file (.mhd) and a raw file (.raw) to the back-end system. The system extracts the metadata and uses it to open the raw image files. The system then uses OpenCV and Numpy to save the image data as image files (.png) and image data arrays (.npy). As the viewers in the gallery are produced, the arrays of images are changed and saved as (.png). This procedure is adopted to avoid nonresponsive changes in the data. Instead, NumPy files are used to construct each image and pass it to the model. This process references the images by their filenames and the NumPy files. This process is elaborated in Fig. (**4**).

##### 3.3.2.3 View CT Scans

The system accesses the back-end picture files and displays them on the front end. For each image, the system sends GET requests to accomplish this. A carousel image or a gallery style can be used to display the photos on the front end. This process is elaborated in Fig. (**5**).

##### 3.3.2.4 Make Predictions

When a user chooses an image from which the forecast is to be done, the system uses the filenames entered by the user during selection to refer to a NumPy file. Prior to feeding this NumPy file to the deep learning model, pre-processing is performed. Following that, the model produces an image mask, as shown in Fig. (**6**).

##### 3.3.2.5 View Predictions

The system uses the related mask and the original reference picture from the CT scan to apply an image contour to the original image. This is shown in Fig. (**7**).

#### 3.3.3 Deep Learning Uses the Convolutional Neural Network Technique to Predict the Lung Cancer

The CNN computation is the most well-known and frequently used one in the domain of DL. The main advantage of CNN over its forebears is that it automatically separates the essential highlights with no human control [10, 27]. Many industries, such as PC vision, conversation handling, face recognition, and others, have extensively used CNNs. Similar to how the traditional brain is organised, neurons in human and animal brains drive the development of CNNs. A complex collection of cells frames the visual cortex in the brain of the feline, and the CNN replicates this succession [18, 33]. Researchers identified three key benefits of CNN [34]: similar depictions, ineffective cooperation, and boundary sharing. In CNN, shared loads and local associations are used to genuinely benefit from 2D information structures like picture signals, in contrast to conventional completely associated (FC) organisations. A minimal number of boundaries are used in this activity, which speeds up the organisation and enhances preparation interaction. This is identical to the cells of the visual cortex. In other words, rather than detecting the entire area, these cells detect small districts (*i.e*., these cells spatially separate the nearby relationship accessible in the information, similar to neighbourhood channels over the information) [8], which is the actual point of concern in the proposed prediction system. A general CNN layer architecture for disease prediction for image data from EHRs is shown in Fig. (**8**). The adoption of the CNN layer architecture in the proposed system includes five layers. *i.e*., input layer, convolution layer, pooling layer, fully connected layer, and output layer.

##### 3.3.3.1 Input Layer

This layer of the convolutional brain is known as the input layer, and it contains image data. The three-layered system takes care of the picture data [10]. The information layer moulds the image aspect into a specific region. For example, if an image of the “28x28=784” calculation is present, it will be converted into a single segment before being handled in the information.

##### 3.3.3.2 Convolution Layer

Since features of the photographs are removed inside the convolution layer, it is also known as the “include extractor layer.” A picture is connected to a convolution layer as a matter of priority in order to carry out the intricate movement that was previously witnessed. The bit difference between the open fields and the channel is registered, resulting in a single whole integer representing the yield quantity. Once again, at the same point, a stride is taken and continued similarly *via* the entire following response field in the compared image data [9]. A comparison communication will be done by the time it covers the whole process.

##### 3.3.3.3 Pooling Layer

The pooling layer completes the convolution-induced reduction in the spatial volume of the image. Two convolution layers help the pooling layer. It could demand significant computational resources to apply layers without applying pooling layers while adding fully associated layers [33]. Therefore, by using max pooling, the spatial volume of an information image is reduced. Max pooling has been used in a single profundity and is downsized to a 2*2 from 4*4.

##### 3.3.3.4 Completely Connected Layer (FC)

Burdens and inclinations are completely linked in the subsequent layer. Neurons in a single layer are paired with subsequent layers [9], and pictures are used to group them into different classes through preparation.

##### 3.3.3.5 Output Layer

It stores one-hot encoded guidelines that are maintained. This layer prediction performance is the output of true prediction by the total number of samples. The percentage of performance is evaluated using (Eq **1**).


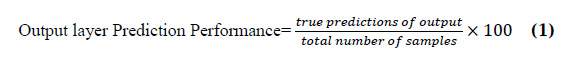


The proposed system includes various stages before passing the EHR image data to the CNN model. It includes image pre-processing, image enhancement, image segmentation, and feature extraction. The proposed system is tested for the prediction of cancer in lung images. Hence, the entire discussion revolves around lung image.

##### 3.3.3.6 Image Pre-processing

We begin the pre-processing stage by improving the quality of the images in order to provide lung image interpretability for human viewers. The pre-processing of images includes division, enhancement, and thresholding. A threshold technique was used to entirely convert the picture to a parallel image [18, 34]. The image quality is totally transferred to the double image by using a global edge. It employs Otsu's method, which slides to alter the limit values in order to reduce or increase the intra-class fluctuation of high-contrast pixels.

##### 3.3.3.7 Image Enhancement

Image upgrading refers to the transformation of an image into better human perception and interpretability of image data. The upgrade method can be used on lung images without destruction, and it is divided into two categories: Spatial domain and frequency domain methodology.

Eq. (**2**) explains the spatial domain processes. In (Eq **2**), f(x, y) is the input image, T is an operator on f that is defined across the area around the point (x, y), and g(x, y) is the output.


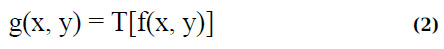


In frequency domain methodology, the pixel is managed to achieve the desired upgrading. In recurrence space, a picture is transferred to recurrence space *via* a Fourier [8] change, which is then reversed to produce the come-about image. The brightness, distinctiveness, and appropriation of the dark level are upgraded in the acquired CT scan image.

##### 3.3.3.8 Image Segmentation

The division outcome typically determines the lung knob for representation and acknowledgement. A threshold division technique approximates the power esteem between the lung knob and the unwanted foundation spot. This leads to the proposed sectioned images and creates a picture [34].

##### 3.3.3.9 Feature Extraction

The prepared work requires the extraction of the knob; hence picture handling algorithms are applied for effective focus on the components of the lung knob. It is then finally possible to discriminate between non-malignant develop- ment and disease in lung images, and the extracted data are highlighted in the data set. Extracted highlights are regarded as the arranging factors and are identified using handling steps and an extraction step in the proposed system.

1) The extraction of the undesired lung region of interest is done using thresholding, locating the region of interest, and then using the freehand method to crop the area by picking it and double-clicking.

2) The extraction of the feature parameters is done by taking the area of interest and applying the classification procedure.

Area refers to the precise number of pixels in ROI, as shown in Eq. (**3**).


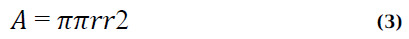


Where r represents the radius.

Diameter refers to the distance separating the circles at the region of interest through the centre of the circle and is evaluated from Eq. (**4**).


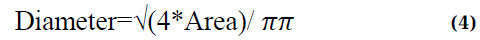


The perimeter is the total distance encircling the area of interest, and it is known by Eq. (**5**).


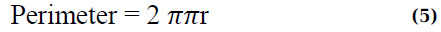


Roundness (eccentricity) refers to how circular the ROI's form is, and it needs to be 1 for circles or less for other shapes. This is evaluated from Eq. (**6**).


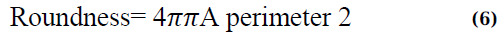


After this volume, the centroid for average intensity is evaluated using Eqs. (**7** and **8**).


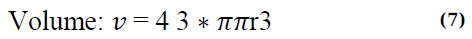



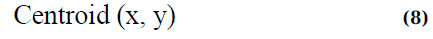


### 3.4 Data Extraction and Prediction

In the proposed system, the information will be taken from the firebase [26], where all user information is the detailed prescriptions kept in the EHR system. Once the data has been extracted, it is processed for the prediction process for early disease diagnosis. To do this, a duplicate data-to-data warehouse was designed to facilitate online analytical processing extracted from a SaaS platform (OLAP). However, as discussed, privacy is one of the challenges to be addressed in healthcare, and therefore, the Firebase access method has been adopted. However, as Firebase access can help us protect data to some extent, this experiment has been conducted and will be kept as a future research problem.

However, large preparation datasets are essential for deep learning because more images can help to improve preparation accuracy. In fact, an unobtrusive measure of information can be more accurate than a poor estimate with a lot of information [35, 36]. But sometimes, an inequity in the classes is another barrier. Calculations involved in deep learning always work best when the number of instances in each class is equal or adjusted to the maximum number. Further, utilizing these images will increase and extend the preparation dataset without adding new images. The first photographs become more varied as the picture count rises, and this is achieved by using turns, flips, interpretations, zooms, and loudness. Thus, the designed deep learning model tries to predict the disease to anticipate it in advance as part of predictive caring.

## 4 RESULT ANALYSIS

The proposed system consists of two modules, namely, an interoperable EHR system and a disease detection system for lung cancer. In this section, a result analysis was performed with respect to the efficiency of both modules. To evaluate the proposed system, a mobile application was developed, and the data were sent to the private cloud for the forbearance of data processing for analysis.

### 4.1 Experimental Setup for EHR System Evaluation

For the evaluation of EHR applications, various instances were considered, as well as the efficiency of the system. First, the EHR system was verified using various instances, such as accumulating data from various hospitals, updating it, deleting it, and analyzing it as per the current content.

#### 4.1.1 Scenario 1

For testing purposes, the major concern is interoperability. The interoperability is tested using instances such as updates and deletion of patient data from various hospitals. The important aspect is to verify the data updates on every patient visit. The updates in interoperability characteristics are explained in a small instance. For example, consider the data updations from Hospital 1(H1). If the patient is new to the system, he/she creates an account in the first visited hospital and updates the information. Soon after the basic credential updates, the health-related data will be updated for the new user. The updations of patient health data from the hospital 1(H1) regarding blood pressure, sugar, height, weight, *etc*., for the patient profile can be seen in Fig. (**9**).

#### 4.2.1 Scenario 2

Let us consider that the patient considered in scenario 1 visits another hospital for another check-up. According to the interoperable characteristics of the EHR system, the data of the patient need to be appended to the previous health record. In Fig. (**10**), the patient profile updations done from hospital 2(H2) are shown for the same patient from scenario 1, with the updated prescription.

From Fig. (**10**), it is very clear that the data are getting appended/updated, and we can confirm the intero- perability characteristics of the proposed system. The same test is repeated for other patient IDs also, and the system provides the same characteristics in the testing phase.

Apart from the interoperable characteristics, the testing of system efficiency is conducted through the existing EPIC system. The EPIC is one of the existing EHR applications, and it is a cloud-based programme that supports several specialties. A wide spectrum of practices employs EPIC, from small hospitals and independent practitioners to multi-specialty hospital groups and hospice care providers. EPIC provides the typical set of “core” EHR functionality, and it allows practitioners to add modules based on their needs. EPIC places a lot of emphasis on promoting remote care and patient interaction. In EPIC, medical records are handled through an EMR (electronic medical records) system. However, the working nature of EPIC EHR is compared with the proposed system to identify the pros and cons. The existingEPICEHRsystemisshown for reference in Fig. (**11**) to show its close working nature.

To compare the systems, the trial version of EPIC and the proposed system were installed in the campus health centre to work together with various users, such as doctors, supporting office staff, lab technicians, and patients. A voting system on the desktop was also triggered for the comparison testing. The comparison testing was based on a user-friendly interface and time. While working with the EPIC, it was found that the EPIC is complex and that no proper interaction is required, as a deficient user-friendly environment was adopted in EPIC. On the other hand, the proposed system was found to be more user-friendly. However, the users can easily understand the proposed system features with a minimum amount of time and work within no time.

To ensure more readability of the proposed system, a *robo* test of the developed application is done; the robo test is an integrated testing tool with the Firebase test. The designed application's user interface (UI) is analysed for structure by *robo* test, which explores its method while automatically mimicking user actions. When a *robo* test is used to test an app on a specific device configuration with the same settings, it always mimics the same user actions in the same order. Thus, a *robo* test validates the fixing of the bug and checks for regressions using a repetitive testing strategy. In order to show the simulated user operations, the *robo* test captures log files, records a series of annotated screenshots, and then produces a film from those screenshots. The tested environment shows failed or passed as a final result, and the same is shown in Fig. (**12**). In the same context, a data analytics test is also performed for the doctor appointment feature. Fig. (**13**) shows the data analytics conducted by the *robo* test for the appointment of data for the period of 14 days.

### 4.2 Result Analysis of Proposed CDSS for Early Prediction of Lung Cancer Using Deep Learning

#### 4.2.1 Scenario 3

For the testing purpose of the proposed CDSS system, which involves deep learning application for the detection of lung cancer, the image data from the EHR system is considered. In this testing, the model is trained to identify whether the lung disease image is cancerous or not. Detection is done using the CNN model, which is expected to yield good results. Here, the model notifies the type of cancer and the available treatments if updated by physicians.

Initially, an effort is made to gather all the information required for the model to classify the images quickly. Many researchers have gathered data from many sources for the implementation and tests using CNN. However, we collected a few samples from valid patients and also from the surrounding health centres. The patient data included an age range of 40 to 70, and both male and female patients.

As we mentioned earlier, an android application has been developed to detect lung diseases. A home page describing the lung disease detection system for the developed application is shown in Fig. (**14**), which performs various operations, such as browsing input images and predicting output,. For the experimentation, “*jpg*” or “*png*” image format is considered rather than “*dcm*” format to fit in the model. While training the system, only three different forms of chest cancer images are considered. The pre-identified images are considered in a separate folder for testing purposes, and the images are classified as (1) adenocarcinoma, (2) large cell carcinoma, and (3) squamous cell carcinoma.

## 5 DISCUSSION

In the process of experimentation, a total of 316 images were considered. In the initial stages, testing was done for 90:10 ratios, and then tested for 80:20 ratios. For evaluation purposes, a manual classification was done for 3 different classes, such as large-cell carcinoma, adenocarcinoma, and squamous-cell carcinoma. Before we proceed further, a brief discussion regarding each disease with appropriate disease detection is as follows:

### 5.1 Large Cell Carcinoma

Large cell carcinoma describes whether lung cancer is there or not. However, many types are in this category, such as large-cell undifferentiated carcinoma, which describes rapid growth and spread throughout the lung. About 10 to 15 percent of patients often have this characteristic. Large-cell undifferentiated carcinoma has a propensity for rapid growth and dispersal. In our experimentation, out of 316 images, large cell images were about 63. Among these 63, 12 random images were picked and tested for identification. Among these, the system was able to achieve an average accuracy of 49.03%. From the description of images in the dataset from healthcare professionals, it was known that the images considered for testing were current or former smokers, with no never-smokers in the cohort. We compared the clinical and proposed predictions, but none of these were significantly different, which is likely due to the limited number of patient images subjected to the study. Fig. (**15**) shows one of the identified large cell carcinomas from the proposed system.

### 5.2 Adenocarcinoma

Adenocarcinoma is another form of lung cancer, where glands in the border of the organs are grown. Lung adenocarcinoma is the most prevalent type of lung cancer, accounting for around 40% of all instances of non-small cell lung cancer and 30% of all cases overall. Breast, prostate, and colorectal cancers are among the frequent malignancies that contain adenocarcinomas. Lung adenocarcinomas are tumours that develop in the mucus-secreting glands that line the outside of the lung. Coughing, hoarseness, weight loss, and weakness are symptoms. For the experimentation of adenocarcinoma identification, a total of 156 images were considered, and 16 images were tested. For this experiment, an average accuracy of about 68.64% was achieved from the proposed system. Fig. (**16**) shows one of the identified adenocarcinoma disease.

### 5.3 Squamous Cell Carcinoma

Squamous cell carcinoma occurs in the middle and outer layers of the skin, which are made up of squamous cells. Squamous cell lung cancer develops in major branches of the airway or in the centre of the lung, where the bigger bronchi connect the trachea to the lung. About 30% of all non-small cell lung cancers are squamous cell lung cancers, and smoking istypically ariskfactor. Fig. (**17**) describes the prediction of squamous cell carcinoma from the proposed system. In the similar grounds of large cell carcinoma, the prediction of squamous cell carcinoma from the proposed system was found to be 71.04% for the total images of 97. It was found that the increase in prediction percentage was due to intense training data received for testing and training for this image type.

Nonetheless, a comparison of the total prediction of different types of lung cancer was made for the proposed system. Fig. (**18**) shows the predictions of different types of cancers from our DSS system for 90:10 training and testing ratios. Among the available 336 images, the prediction percentage for large cell carcinoma, adenocarcinoma, and squamous cell carcinoma was calculated. However, due to fewer sets of training data, the prediction of large cell carcinoma was less compared to adenocarcinoma and squamous cell carcinoma. Apart from the regular analysis, it was found that large cell carcinoma majorly occurs in males due to smoking; however, this cancer is found as breast cancer in females.

Apart from the above analysis, we compared the working of an Android application developed for lung disease detection with the web application developed earlier in the proposed work [37]. The interoperability nature of both applications wasanalogousand foundto function similarly while importing and exporting the EHR data for the early detection of diseases. In earlier work [37], we defined the decision support system using text-based data for diabetic prediction. However, in the proposed work, an attempt was made to train the decision support system using deep learning techniques for lung cancer. Deep learning is necessary for vigorous training and for analyzing image data compared to text data. Both the systems performed well with respect to the *robo*-test and accuracy test. However, while evaluating the accuracy, many parameters were assumed, where the True Positive (TP) is the successfully predicted positive value, implying that both the actual and expected class values are true. The True Negatives (TN) are the accurately predicted negative values, indicating that neither the actual class value nor the projected class value is positive. The False Positives (FP) are the values when the anticipated class is true, but the actual class is false. The False negatives (FN) are the values when the anticipated class is false, but the actual class is true. From these parameters, the accuracy of the proposed system was evaluated. When the test across the parameters on the split datasets was carried out, varied levels/scores in accuracy parameters were found. The reason for this may be due to a number of elements present in different age groups. Hence, after the confirmation of the well-behaving algorithm, an analysis was performed with respect to the age groups present in the standard dataset. The dataset had entries from the age of 40 to 70 years. However, for accurate analysis, an age group of 40 to 50, 51 to 60, and 61 to 70 was performed.


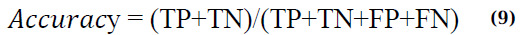


Eq. (**9**) is used to evaluate the accuracy score, which is the most instinctive performance measure, and the ratio of truly predicted observations to the total observations. A bar graph plotted in Fig. (**19**) shows the accuracy levels of the earlier and the proposed system with respect to the different age groups [37]. From the disease prediction of the proposed system, we can conclude that CNN contributed more than the ANN method for various age groups, such as 40 to 50, 51 to 60, and 61 to 70 [37].

## CONCLUSION

As the challenges are increasing daily in healthcare industries, a secure, interoperable EHR was designed to help patients and doctors access patient data efficiently and effectively using Android applications. Further, a decision support system using a deep learning model was designed successfully. Early disease detection for lung cancer was evaluated, and the model achieved an accuracy of 93%. Additionally, the integration of EHR data was required to identify the causes through deep learning methods for the early detection of various diseases. In this way, it was found that non-smokers are more prone to cancer than smokers [30, 38]. Hence, there is a need to investigate this area of research further.

## AUTHORS’ CONTRIBUTIONS

It is hereby acknowledged that all authors have accepted responsibility for the manuscript's content and consented to itssubmission. They have meticulously reviewed all results and unanimously approved the final version of the manuscript.

## Figures and Tables

**Fig. (1):**
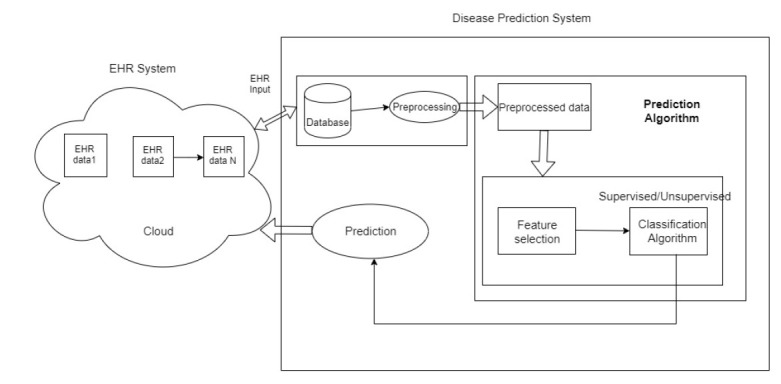
An overview of the proposed system.

**Fig. (2):**
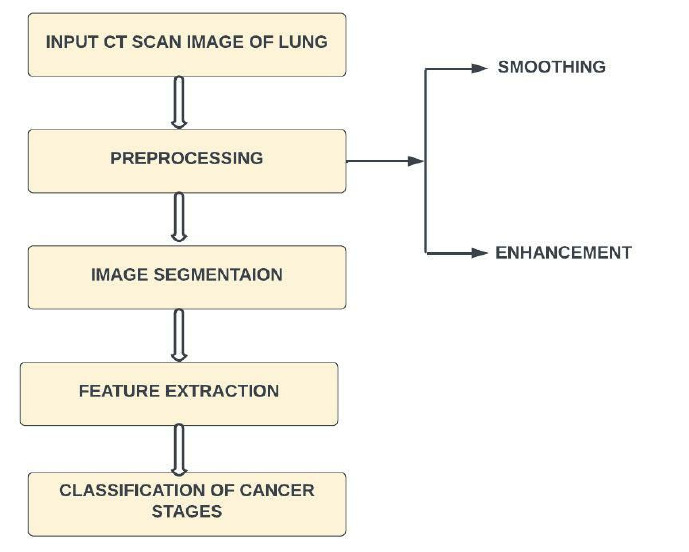
Overview of using deep learning for lung disease detection.

**Fig. (3):**
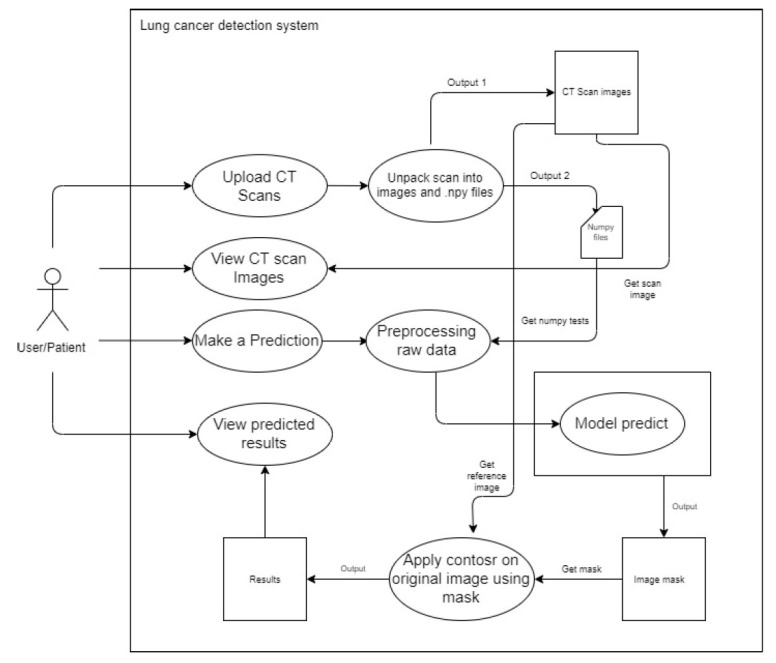
Overview.

**Fig. (4):**
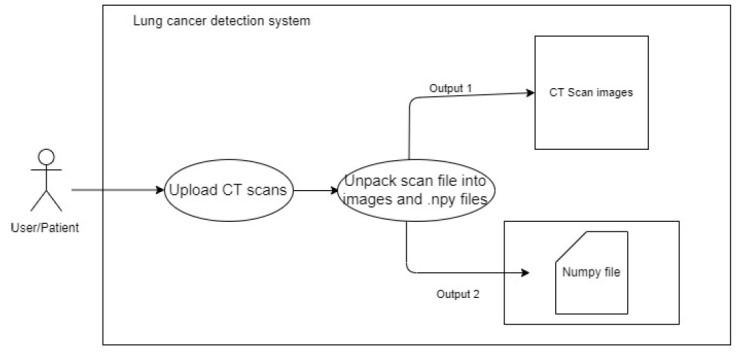
To upload CT scan image.

**Fig. (5):**
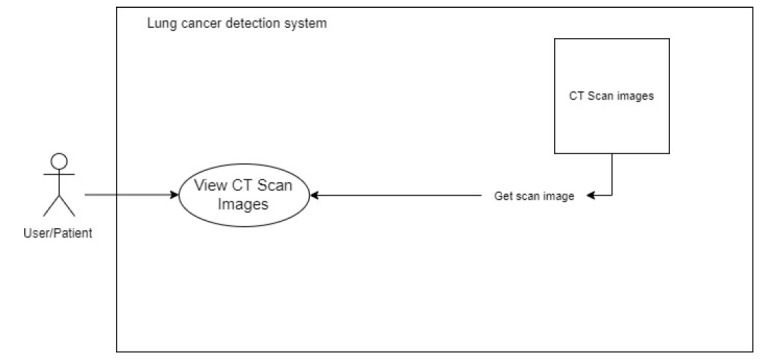
To view the CT scan image.

**Fig. (6):**
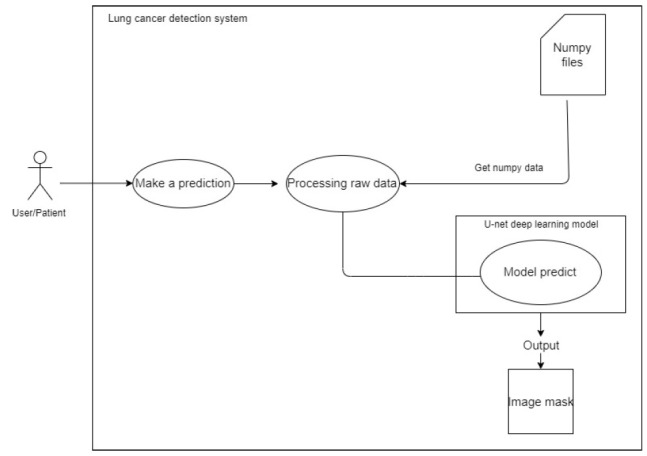
For making predictions.

**Fig. (7):**
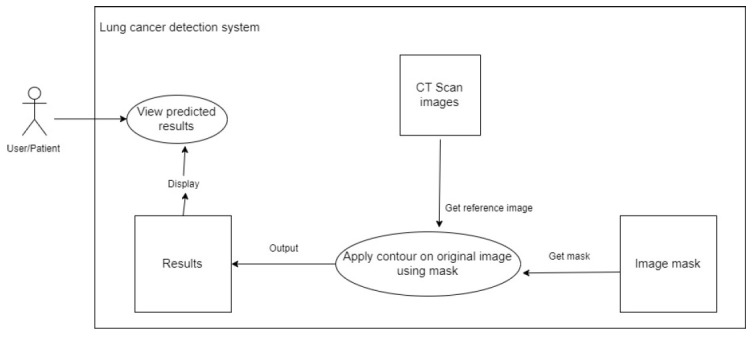
To view predictions.

**Fig. (8):**
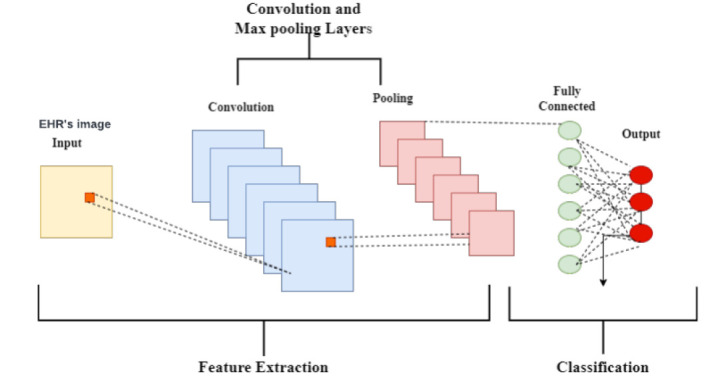
CNN layer architecture.

**Fig. (9):**
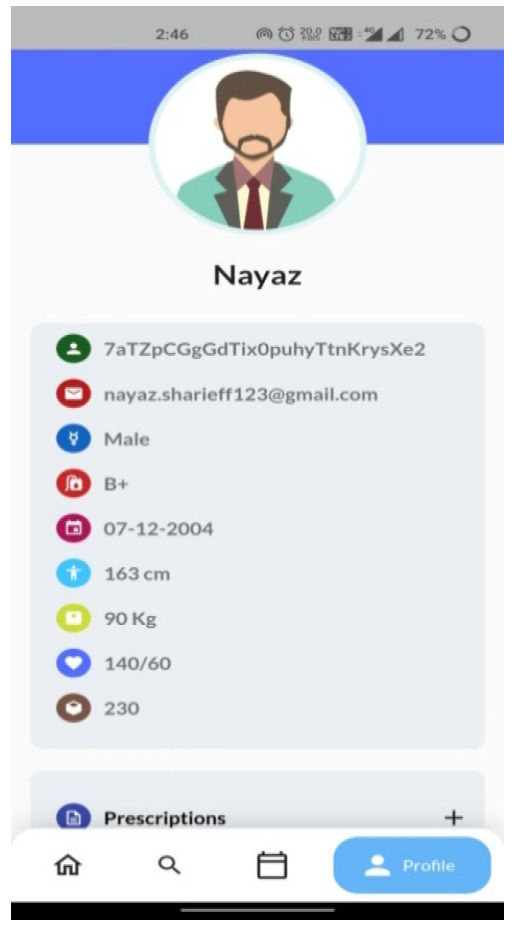
Patient profile from H1 hospital.

**Fig. (10):**
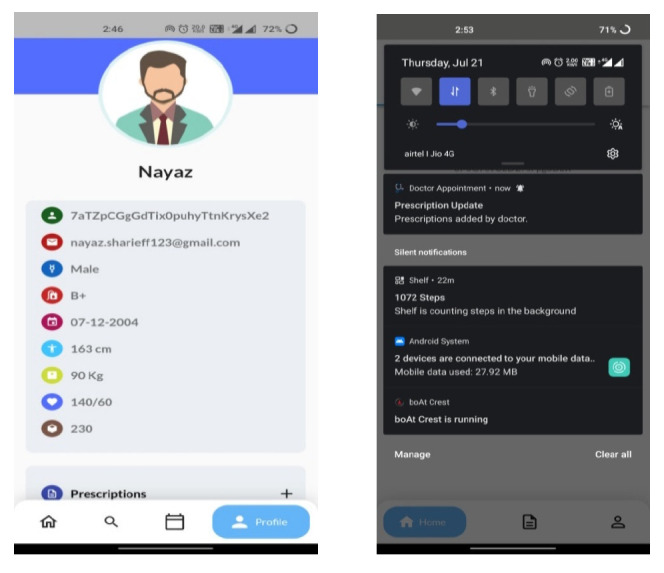
Patient profile from H2 hospital with a prescription update.

**Fig. (11):**
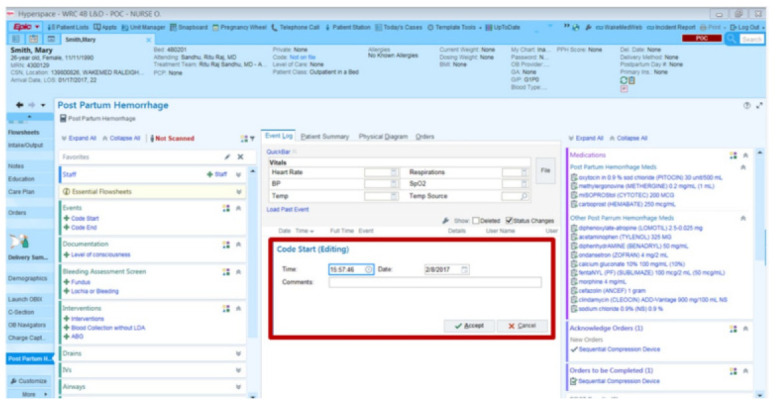
EPIC EHR software.

**Fig. (12):**
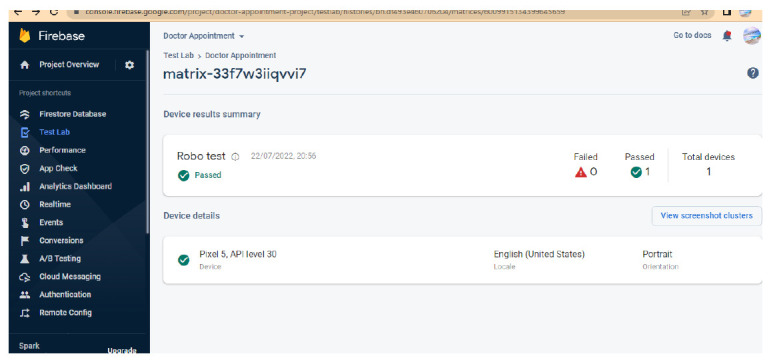
Robo test of android application.

**Fig. (13):**
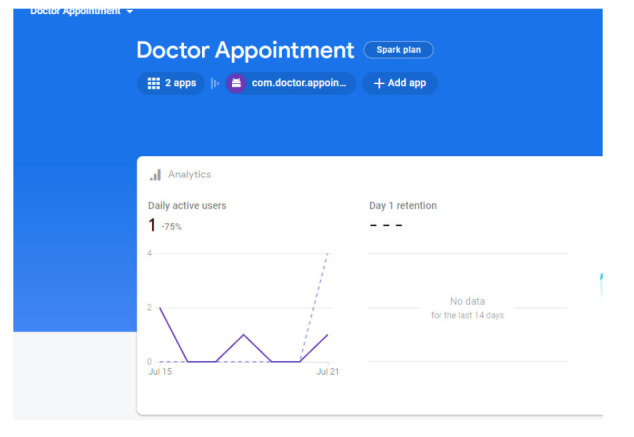
Analytics of doctor application.

**Fig. (14):**
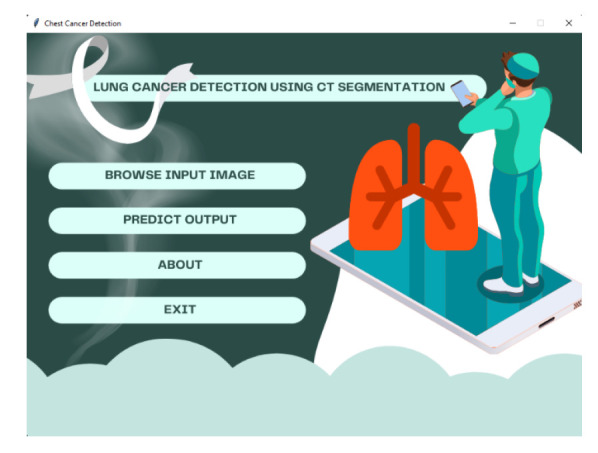
Home page of the lung disease detection of the application.

**Fig. (15):**
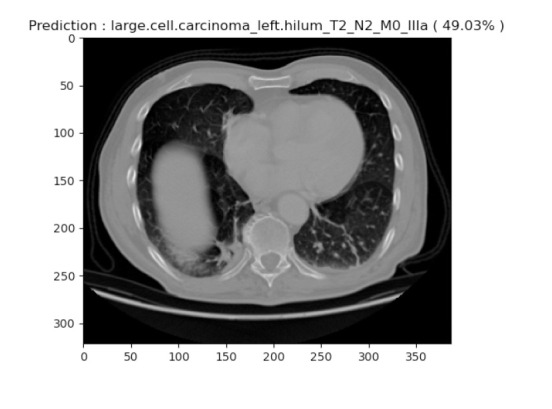
Prediction of large cell carcinoma.

**Fig. (16):**
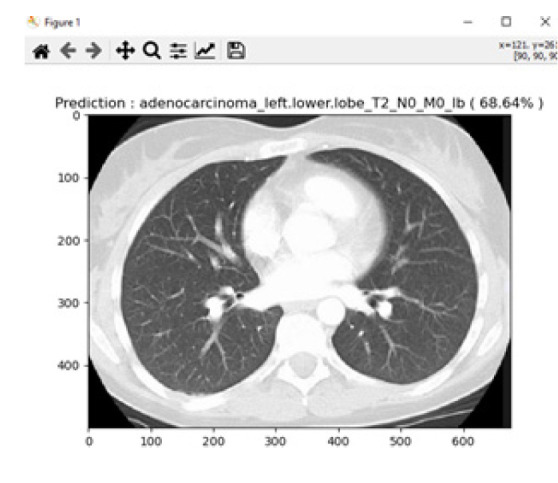
Prediction of adenocarcinoma.

**Fig. (17):**
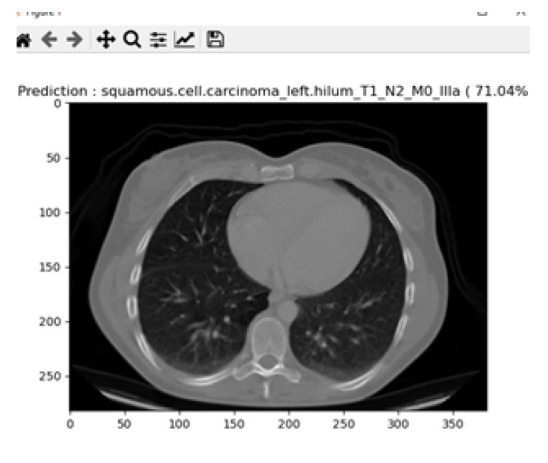
Prediction of squamous cell carcinoma.

**Fig. (18):**
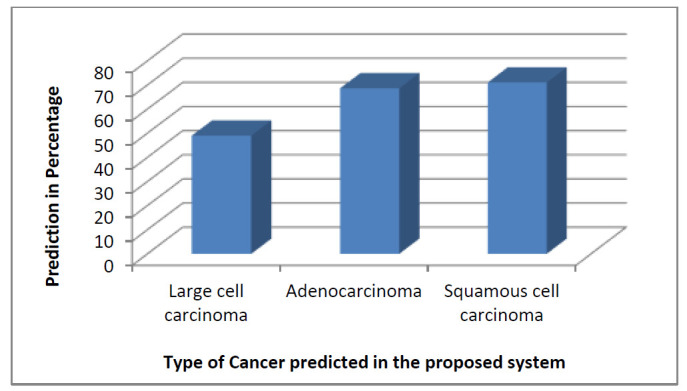
Different cancer type prediction for 90:10 training and testing ratios.

**Fig. (19):**
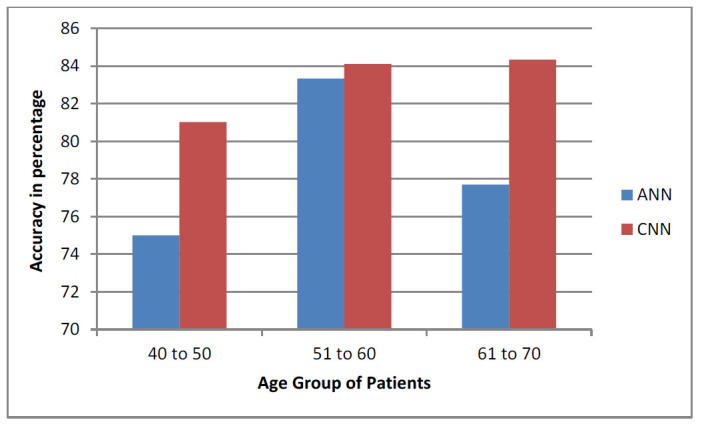
Accuracy performance of earlier system [37] and prosed system.

**Table 1:** Advantages of having health records in the form of EMR.

**S. No.**	**Advantages**	**Description**
1	Easy record maintenances	Standard, more efficient record-saving for lab findings, staff assessments, and other documents like expert notes.
2	Effectively recovered	All authorized members of the medical care team can approach the patient's records indefinitely.
3	Error-free	Reducing errors caused by illegible writing or records can be avoided.
4	Security	Built-in security and protection features in EMRs ensure that only authorised individuals have access to sensitive patient data.

**Table 2:** Challenges of having health records in the form of EMR.

S.No.	Challenge	Description
1	EMR is breaking the financial plan	It is obvious that the expense of buying the software is high and it is tremendous to introduce EMR. This incorporates equipment, programming, execution help, preparation, support, and ongoing organization expenses. The expense of EMR is the main reason numerous medical care organizations have not yet switched from paper-based record maintenance to electronic documents [6].
2	EMR does not fit with the work process	Some clinical foundations might find that, even after cautious preparation and execution, EMR simply does not give the usefulness they need. Envision a situation where you have put a significant measure of cash into the innovation yet can not utilize it to its fullest potential [7].
3	Training employees is difficult	Scheduling EMR instructional meetings and arrangements for key representatives to commit their time and work to have a ton of experience with EMR, who can act as ministers for innovation and as an asset for doctors and other staff individuals [8].
4	Physicians resist using EMR	Some doctors essentially decline to acknowledge new innovation, either because they do not trust its capacities [9] or maybe they are threatened by its range. They feel a distributed methodology will work best in this present circumstance.
5	EMR does not meet meaningful prerequisites usage	It is challenging to stay aware of guidelines, which are precisely to confirm meaningful usage [9, 10].

**Table 3:** Benefits of EHR.

S. No.	Benefit	Descriptions
1	Efficient record accessibility	EHRs ensure that patient records are efficiently accessible from anywhere, at any time, and through any communicable resources.
2	Efficient storage	EHRs may be stored efficiently, with little space requirements, and indefinitely.
3	No loss	EHR loss is not possible because of the effective organization of computer network applications.
4	Cost-effective	EHR is financially astute and works with the nature of patient records.
5	Consistency	EHRs help track patients' treatment progress and promote ongoing consistency.
6	Consolidated	EHR provides a summary report of all people’s clinical encounters.
7	Fast	EHRs work to improve analysis speed and precision and avoid repetition of tests.
8	Efficient sharing	EHRs can be efficiently transported inside and between medical offices.
9	Multi-user	EHRs can be made available by multiple clients at the same time and are easy to update.
10	Backup and recoverability	EHRs allow for the holding of backup copies of patient records at a meager cost.
11	Evidence-based care	EHRs provide evidence-based care and work with more advanced medical service options.
12	Academic	EHRs can be used for academic research.

**Table 4:** Challenges in EHR usability and design.

S. No.	Challenges	Descriptions
1	Interoperability	Interoperability of EHR needs efficient design issues
2	Implementation cost	Implementation cost of the EHR is high compared to EMR due to network application development and maintenance.
3	Staff resistance	Staff resistance due to new technology advancement is one of the hurdles to implementation in real scenarios.
4	Training	Training the staff is time-consuming if they are not well-educated about the use of the software.
5	Usability	Lack of usability requires proper infrastructure.
6	Data privacy	Data privacy is compromised due to negligence of security and trust issues while sharing and accessing the records in the EHR system.

**Table 5:** Features of deep learning.

S. No.	Features	Descriptions
1	Universal learning approach	DL can act in all application areas, and it is alluded to as general learning.
2	Robustness	As a general rule, unequivocally planned highlights are not needed in DL methods. All things being similar, the advanced elements are learned in a computerized design connected.
3	Generalization	Different information kinds or applications can use a similar DL approach, which is frequently referred to as “transfer learning” (TL), as explained in the previous segment. Additionally, it is a helpful methodology for problems with a shortage of information.
4	Scalability	DL is incredibly scalable, as there are no restrictions on its use and organization. ResNet, a Microsoft invention, has 1202 layers and is frequently used at a supercomputing scale. A similar methodology was adopted by Lawrence Livermore National Laboratory (LLNL) for creating systems for networks. This methodology allows for the execution of several hubs.

## Data Availability

The data and supportive information are available within the article.

## LIST OF ABBREVIATIONS

EHRs: Electronic health records
CNN: Convolution Neural Network
DCT: Data and communications technology
EMRs: Electronic medical records
CPR: Computer-based patient record
ONC: Office of the National Coordininator
DSS: Decision support system
AIIMS: All India Institute of Medical Sciences
PGIMER: Postgraduate Institute of Medical Education and Research
CHISTAR: Cloud Health Information Systems Technology Architecture
CT: Computed Tomography
PSO: Particle Swarm Optimization
